# Effect of Regular Resistance Training on Motivation, Self-Perceived Health, and Quality of Life in Previously Inactive Overweight Women: A Randomized, Controlled Trial

**DOI:** 10.1155/2016/3815976

**Published:** 2016-07-04

**Authors:** Hege Heiestad, Anne Mette Rustaden, Kari Bø, Lene A. H. Haakstad

**Affiliations:** Department of Sports Medicine, Norwegian School of Sports Sciences, 0863 Oslo, Norway

## Abstract

*Objectives*. The aim was to investigate the effects of three different types of resistance training implementation.* Design*. Randomized controlled trial.* Methods*. Inactive, overweight women (*n* = 143), mean BMI 31.3 ± 5.2 kg/m^2^, mean age 39.9 ± 10.5 years, were randomized to one of the following groups: A (BodyPump group training), B (individual follow-up by a personal trainer), C (nonsupervised exercise), or D (controls). The intervention included 12 weeks of 45–60 minutes' full-body resistance training three sessions per week. The outcomes in this paper are all secondary outcome measures: exercise motivation, self-perceived health, and quality of life.* Results*. Adherence averaged 26.1 ± 10.3 of 36 prescribed sessions. After the intervention period, all three training groups (A–C) had better scores on exercise motivation (A = 43.9 ± 19.8, B = 47.6 ± 15.4, C = 48.4 ± 17.8) compared to the control group (D) (26.5 ± 18.2) (*p* < 0.001). Groups B and C scored better on self-perceived health (B = 1.9 ± 0.8, C = 2.3 ± 0.8), compared to group D (3.0 ± 0.6) (*p* < 0.001). For quality of life measurement, there was no statistically significant difference between either intervention groups or the control.* Conclusions*. Resistance training contributed to higher scores in important variables related to exercise motivation and self-perceived health. Low adherence showed that it was difficult to motivate previously inactive, overweight women to participate in regular strength training.

## 1. Introduction

Over the last three decades, fitness centers have become a large and growing area for physical activity and exercise [1]. Worldwide, it has been estimated that the fitness industry has about 145 million members and more than 180 000 centers/gyms and generates about 84 billion dollars [2]. For the past 20 years, the number of fitness centers and number of members have increased also in Scandinavia. In 2009, almost one-third of the adult population in Norway reported that they were members of a fitness center, compared to 8% in 1987 [1].

Even though health clubs and fitness centers have become a large and growing venue for physical activity and exercise, the scientific knowledge about the effect of various fitness concepts on different health outcomes is sparse. Search on PubMed revealed only three randomized controlled trials (RCTs) evaluating the effect of regular resistance training in fitness centers either performed in a group training setting (BodyPump) [3] or supervised by a personal trainer [4]. The studies scored low on methodical quality according to the CONSORT Statement checklist [5] and did not measure the impact of regular exercise on psychological variables or mechanisms supporting the positive effects of exercise on mental health. Hence, scientific evidence is still limited on the effect of widespread and common training concepts. Thus, the aim of the present study was to evaluate the effects of three different types of resistance training implementation. Primary outcomes of the overall trial were maximal (1RM) and submaximal (70% of 1RM) muscle strength. The outcomes in this paper are secondary outcome measures, including exercise motivation, self-perceived health, and quality of life.

## 2. Methods

### 2.1. Study Design and Population

This was a single-blinded, randomized controlled trial with the primary aim of comparing the effect of three different resistance training concepts on muscle strength and body composition, conducted at the Norwegian School of Sports Science in Oslo in 2012/2013. The participants were recruited via social media such as Facebook and by posters distributed in the local environment. At first phone contact, the aims and implications of the study were explained and the eligibility criteria were checked. Overweight women (BMI ≥ 25), between 18 and 65 years of age, previously nonexercisers, defined as not performing regular exercise ≥ once a week, as well as ability to speak and understand Norwegian, were eligible for the trial. All eligible participants confirmed orally that they were motivated to partake in the study and the exercise intervention. Exclusion criteria were being pregnant at the time of study start, participation in a similar research project, planned vacation/absence > 2 weeks during the intervention period, women who expressed low likelihood for regular exercise participation, and/or disease/injury with contraindications for regular physical activity. If there were any uncertainty about participation due to health reasons, the current participants were asked to get verbal approval from their doctor.

Prior to baseline testing, an e-mail describing the purpose and background of the study was sent to the participants. All women gave written consent to participate, and the Regional Committee for Medical and Health Research Ethics, Southern Norway, approved the study (REK 2012/783). The project was completed in accordance with the CONSORT Statement [5], and the procedures followed the World Medical Association Declaration of Helsinki. The study is listed in the ClinicalTrials.gov (NCT01993953).

A priori sample size calculation was done for the primary outcome (muscle strength), showing that a minimum sample size of 30–35 participants per group (in total 120–140) was required to detect 11% difference in muscle strength (standard deviation of 15%) at the 0.05 level, with a power of 0.80 [3].

Out of 195 women who contacted the primary investigator, 143 fulfilled the inclusion criteria and were randomized to four different groups: A: BodyPump, B: follow-up by a personal trainer, C: nonsupervised exercise, or control group (D). Hence, each group consisted of 35 or 36 women. Allocations were sealed in opaque envelopes following a simple computer-based randomization program.

The participants were requested not to reveal group affiliation, and all assessors were blinded to the participant's allocation while testing and assembling questionnaire data, as well as plotting and analyzing the data.

### 2.2. Intervention

Women randomized for exercise (groups A–C) were informed to participate in three 45–60 minutes' resistance trainings sessions weekly, for a minimum of 12 weeks. Group A (BodyPump) received a 12 weeks' membership at about 20 elective SATS-fitness centers in Oslo or Akershus, where they followed ordinary BodyPump classes, scheduled for all the members at the fitness center. The instructors were not asked to change their normal classes in any way. Each session started with 5-6 minutes of warm-up, followed by a high repetition resistance full-body workout program (30–112 repetitions), with no resting periods. The training program included legs (squats, lunges), chest (bench press), back (dead rows, dead lifts), triceps (triceps press), biceps (biceps crunches), shoulders (push-ups, shoulder raises, rowing, and shoulder press), and abdominals and core (sit-ups, planks, and crunches). The last 5 minutes contained cool-down and stretching. The participants selected their resistance weight load with respect to the available plates (the weights of the plates were 1.25, 2.5, 5, and 10 kg and could be increased incrementally as needed).

The resistance training program for groups B and C was designed and structured to be comparable to the BodyPump concept but differed with respect to the total weight load, number of repetitions (3–15), series (1–4), and pauses (30 seconds–2 minutes). It was a nonlinear periodization-program, divided into three different sessions each week in the range of 60–90% of repetitions maximum. Session 1 (“medium” intensity, 70–80% of 1RM) included 8–10 repetitions, 2–4 series, and pauses of 60 seconds. Session 2 (“light” intensity, 60% of 1RM) included 13–15 repetitions, 2–4 series, and 60 seconds' pauses as well, while session 3 (“hard” intensity, 80–90% of 1RM) included 3–6 repetitions, 2–4 series, and pauses of 120 seconds.

For group B, a personal trainer was present and provided the women with one-to-one supervision, ensuring that all exercises were performed correctly with low risk of injuries as well as “spotting” when needed. “Spotting” in resistance training is the act of supporting another person during a particular exercise, with emphasis on allowing the participant to lift or push more than they could normally do safely [6]. Group C received the same resistance program as group B but were only given instructions at their first and 18th session of in total 36 sessions. Otherwise, the program was conducted individually.

The controls (D) were informed to maintain the same lifestyle and dietary habits as before and were neither encouraged nor discouraged to exercise. There was no financial compensation to the participants.

Prior to the intervention period, all groups (A–D) received a diary to record exercise participation, including frequency, duration, and intensity. The purpose of giving an exercise diary to the control group was to identify possible changes in activity pattern during the intervention period. The exercise diaries were handed in at posttest.

### 2.3. Assessment Procedures and Outcome Measures

A standardized questionnaire was filled out at baseline and after the intervention, including questions adopted from the Behavioural Regulation in Exercise Questionnaire-2 (BREQ-2), SF-36, and Satisfaction With Life Scale (SWLS), all three considered to be reliable and valid instruments to measure exercise motivation (BREQ-2), self-perceived health (SF-36), and quality of life (SWLS) [7–11]. A pretest of the questionnaire was conducted prior to the present study to ensure face validity, and five women were asked to evaluate question sequence, continuity, length, and timing. A few changes were made, such as reformulating some questions and statements.

The final questionnaire was self-filled, contained 79 questions in total, and required roughly 15 to 20 minutes to answer. In addition to questions related to exercise motivation, self-perceived health, and quality of life, the women reported on different demographic variables. Data were collected at the Norwegian School of Sports Sciences. The main researcher was present and available to answer questions from the participants at baseline and posttest.

The outcome measures were exercise motivation, self-perceived health, and quality of life. Exercise motivation was measured with the BREQ-2 [10]. The questionnaire has been used in a number of studies with different study populations, including overweight adults [12], and is considered to be a valid assessment instrument to measure reasons underlying an individual's drive to engage or not to engage in physical activity [13]. BREQ-2 included 19 statements divided into 5 different subscales, where the participants rated their answers from 0 (not true for me) to 4 (very true to me). The questionnaire measures five different subscales of motivation from amotivation at one end of the scale (lowest level of self-determination) through various forms of external motivation to internal motivation at the other end of the scale (highest level of self-determination). It is based on the assumption that internal-external versus controlled motivation creates different psychological readiness to sustain increased physical activity [14]. According to BREQ-2, the responses were analyzed as five subscales and a total sum score, Relative Autonomy Index (RAI). RAI is obtained by applying a weighting to each subscale and then summing these weighted scores, RAI_BREQ-2_ = ∑([amotivation  *x* − 3] + [external regulation *x* − 2] + [introjected regulation *x* − 1]+[identified regulation *x* + 2] + [intrinsic regulation *x* + 3]), and provides a measure of the overall level of a person's exercise motivation [14].

Self-perceived health was assessed with two questions obtained from the Norwegian version of SF-36 [8]. Question  1* “In general, would you say your health is…”* was rated from 1 = “excellent” to 5 = “poor,” and question  2* “Compared to one year ago, how would you rate your health in general now?”* was rated from 1 = “much better now than one year ago” to 5 = “much worse now than one year ago” [11].

Quality of life was measured by the Norwegian version of the Satisfaction With Life Scale (SWLS). According to the SWLS, the women rated their feelings regarding five different statements on a 7-item scale (1 = strongly disagree to 7 = strongly agree) to assess satisfaction with life:* “In most ways my life is close to my ideal,” “The conditions of my life are excellent,” “I am satisfied with my life,” “So far I have gotten the important things I want in life,”* and* “If I could live my life over, I would change almost nothing.”* The results were analyzed separately and as a sum score. According to Diener et al. [7], a total score of 20–24 is considered to indicate an average level of life satisfaction, while a score ≥ 31 or higher is associated with being “extremely satisfied with life.”

### 2.4. Statistical Analysis

Statistical Package for the Social Sciences (SPSS), 19.0, for Windows was used for all statistical analysis. Data were presented as numbers (*n*) with percentages or means with standard deviations (SD). Level of statistical significance was set at *p* < 0.05. Posttest results are reported for completers only (*n* = 90). The Shapiro-Wilk test was used to analyze if the data were normally distributed. Potential differences between the four groups (A–D) with respect to our outcomes were analyzed by one-way ANOVA and Tukey's post hoc test [15].

## 3. Results

**Figure 1:**
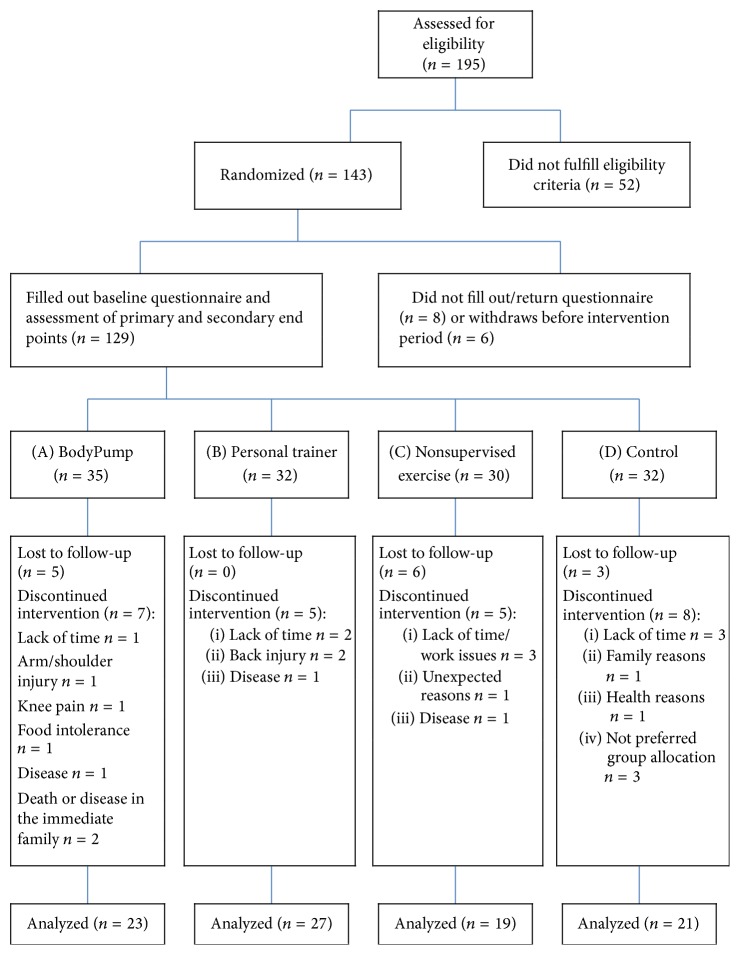
Trial profile showing the flow of participants through the RCT.

Out of 143 participants meeting the inclusion criteria, 129 (90.2%) responded to the questionnaire at baseline (Figure 1). The women came from the city of Oslo and were all of Norwegian descent. The average age was 39.9 ± 10.5 years and mean BMI was 31.3 ± 5.2 kg/m^2^. The majority (70.6%) of the participants were working full time, five women (3.5%) worked less than 50%, and three women (2.1%) were sick-listed.

There were no statistically significant differences between the groups in demographic variables (age, weight, height, BMI, or employment percentages) at baseline (Table 1) or outcome variables except from the statement* “In most ways my life is close to my ideal”* for quality of life, where group A (BodyPump) scored significantly higher compared to group D (control) (*p* = 0.02) (Table 2).

A total of 53 women were lost to follow-up, with 14 not completing baseline evaluations and 39 not showing up at posttest.

Figure 1 shows the trial profile and the flow of participants throughout the study period, including reasons for dropouts. There were only small differences in causes of dropouts between the four groups.

Mean adherence to the exercise protocol was 26.1 ± 10.3 out of 36 prescribed exercise sessions, with the following distribution in groups A (BodyPump), B (personal trainer), and C (nonsupervised exercise): 20.7 ± 7.8, 33.2 ± 4.3, and 27.8 ± 6.6, respectively. Only eight women (7.4%) had 100% exercise adherence and participated in three sessions weekly, with the highest attendance in group B (personal trainer, *n* = 6). Forty women (37.0%) participated in at least two sessions weekly. Adherence rates are based on registrations in the exercise diary, submitted by 63 participants in groups A–C at posttest. In group D (control), seven women handed in their exercise diary; four of them were not filled out, and three women had done some exercise (walking, yoga, and endurance training in ellipse-machine) during the intervention period.

Table 3 shows the results for outcome variables after the exercise intervention. All three training groups (A–C) had higher scores on exercise motivation compared to the control group (D), with respect to total sum score (RAI) (*p* < 0.001), as well as the subscales introjection (*p* < 0.001), identified (*p* < 0.001), and integrated regulation (*p* < 0.001). In addition, groups B (personal trainer) and C (nonsupervised exercise) had significantly better results on one of the questions related to self-perceived health (*p* < 0.001). There were no statistically significant differences between the four groups in the outcome variable quality of life, assessed by SWLS.

## 4. Discussion

This is one of few RCTs to investigate the effects of three different types of resistance training implementation, including exercise motivation, self-perceived health, and quality of life. The main findings of this study showed that all training groups (A–C) had higher scores in important variables related to exercise motivation and self-perceived health with the best results in group B (personal trainer). Unfortunately, the adherence to the exercise program was low. According to several studies, being overweight is considered to be one of the main predictors of attrition from regular exercise [16, 17]. It is therefore important to develop strategies to promote exercise adherence in overweight and obese individuals. In the present study, the use of a personal trainer seemed to support compliance and contributed to significantly higher levels of exercise adherence compared to both the unsupervised training and the group training.

The strengths of the present study were use of a RCT design, clear inclusion and exclusion criteria, blinding of the assessors, and prespecified primary and secondary outcome measures. Study limitations included the sample size, which was not based on a priori power calculations for our variables, high loss to follow-up at posttest, and low adherence to the exercise protocol. Adherence to exercise was only monitored by self-recording, which may have led to overestimation [18].

A dropout rate of 30% has reduced the power of the study and the ability to draw clear conclusions. Post hoc power calculation based on the present results showed that, with 80% power and *p* < 0.05, at least 45 women would be needed in each group (totally 180 participants) to show statistically significant differences in our outcomes. Hence, future studies may use these numbers to estimate required sample size when planning a RCT evaluating the effect of exercise on these variables.

One method commonly used to analyze incomplete dataset is to analyze the participants who completed all tests and follow-up procedures only [19]. However, such analyses are often biased as the dropouts may have a tendency to be different from participants following research protocols and prescribed exercise programs [20]. Further bias is present if the dropout rates differ between the intervention groups [20]. In the present study, group B (personal trainer) had about 16% dropout, while groups A (BodyPump), C (nonsupervised exercise), and D (control group) had about 35% dropout. Herbert et al. [21] have emphasized that measures of key outcomes should be obtained from >85% of the participants, as imputation techniques can never reproduce missing data. Hence, using the Last Observation Carried Forward (LOCF) method to achieve a complete dataset would not be suitable for analyzing the current results [21].

At first phone contact, women who expressed low likelihood for regular exercise participation were excluded. Hence, all eligible participants stated that they were motivated to partake in the study and the exercise intervention. Why the women in the present study did not adhere is difficult to interpret, and information about reasons for the low participation rate is unfortunately not available.

Also previous trials in sedentary overweight women have reported low adherence to the exercise program or have not reported adherence at all [22].

It is possible that the rigid obligation to comply with a minimum of three exercise sessions weekly was difficult to adhere to, especially for previously nonexercisers. According to Biddle and Mutrie [23], exercising twice a week for untrained individual might be sufficient to increase psychological factors such as motivation, inspiration, emotional strength, and general wellbeing. In our study, 37.0% participated in at least two sessions weekly, compared to 7.4% exercising according to protocol. However, analysis of the data for the subjects who attended at least twice a week did not change the results. Small number unfortunately limits this analysis. Future research is required to investigate optimal dosage and mode of activity in different populations, including overweight and obese individuals.

A general perception is that exercising with a personal trainer is more beneficial for improving health-related fitness than training with a group or alone. However, there is little evidence confirming these assumptions, and the results of the present study did not show better results in the outcome variables with the personal trainer (group B). On the other hand, adherence was highest in the personal trainer group, with a total of 33 out of 36 sessions. Hence, training with a personal trainer seemed to increase the participant's motivation. One explanation for this may be that they were given specific feedback and follow-up on their technique and performance. This corresponds with the literature indicating that a sense of autonomy, social influence, and knowledge are important elements in terms of maintaining motivation for exercises and long-term lifestyle changes [24–26]. According to Levinger et al. [27] it is easier to regularly attend sessions and also to work harder with personal motivation and supervision. A limitation of the study is that the outcome variables did not capture the characteristics predictive of adherence as self-efficacy, self-esteem, and body image which would have been highly relevant yet were not measured or reported.

Understanding motivation for engaging in physical activity and exercise has been a key issue in sports medicine and science [28]. At posttest, measures of five different subscales of motivation showed that groups A–C were significantly more motivated and had higher intrinsic motivation compared to women being controls (D). This may be an important finding. Former nonexercisers who participate in an exercise program can increase their motivation and confidence to continue exercising after intervention. Other studies have confirmed that intrinsic motivation (enjoyment, pleasure, and challenge) may be one of the primary sources for the individual's actions and that its presence facilitates behavioural maintenance and adherence [29]. Hence, high intrinsic motivation is likely to increase regular exercise participation [30].

We found significant differences in self-perceived health in response to the question* “Compared to one year ago, how would you rate your health in general now?”* where group B (personal trainer) and group C (nonsupervised exercise) scored higher compared to the control group (D). We included only two out of 11 questions adapted from the short version of SF-36 [8], but these two questions are similar to the simple five-point scale self-rated heath question, recommended by World Health Organization (*“How is your health in general,”* with the possible response options 1 = “very good” to 5 = “very bad”) [31]. Intentionally, the question is formulated to be vague and the purpose is to assess the individuals own classification of health, including mental and social dimensions. Several studies have shown that this question to assess self-rated health is a statistically powerful predictor of mortality in all populations [31, 32].

A high mean value for quality of life at baseline may have caused a ceiling effect and contributed to smaller improvements in scores at posttests [33]; due to that a scale from 1 to 7 may not be sensitive enough to detect significant differences between individuals.

## 5. Conclusion

Regular resistance training for 12 weeks in previously inactive, overweight women contributed to higher scores in important variables related to exercise motivation and self-perceived health compared to a control group after the intervention. The best results were achieved by a tailored exercise program and individual follow-up by a personal trainer. In the present study, the use of a personal trainer contributed to higher levels of exercise adherence, compared to group training and exercise performed individually.

## Figures and Tables

**Table 1:** Background variables for the different training groups A–C and control group D (*n* = 129). Results are shown as mean and standard deviation (SD).

Outcome measures	Group A	Group B	Group C	Group D	*p* value
	BodyPump (*n* = 35)	Personal trainer (*n* = 32)	Nonsupervised exercise (*n* = 30)	Control (*n* = 32)	
Age (years)	38.1 (10.8)	38.4 (9.3)	41.1 (10.5)	42.2 (10.2)	0.360
Weight (kg)	85.5 (13.9)	91.9 (21.1)	87.7 (13.2)	88.4 (16.2)	0.500
Height (cm)	168.3 (5.9)	169.0 (6.2)	168.0 (6.5)	168.1 (5.5)	0.919
BMI (kg/m^2^)	30.2 (5.0)	31.9 (6.1)	31.1 (4.5)	31.3 (5.2)	0.667
Work hours (%)	85.4 (29.1)	93.1 (23.5)	89.7 (32.9)	91.8 (26.5)	0.694

**Table 2:** Primary and secondary outcome measures for completers in the different training groups A–C and the control group D (*n* = 90) analyzed by one-way ANOVA at baseline. The results are shown as mean and standard deviation (SD).

Outcome measures	Group A	Group B	Group C	Group D	*p* value
	BodyPump (*n* = 23)	Personal trainer (*n* = 27)	Non-supervised exercise (*n* = 19)	Control (*n* = 21)	
*Exercise motivation (ranked from 0 to 4, where 0 represents not true for me and 4 represents very true for me, total score calculated for each individual subscale and for Relative Autonomy Index (RAI)):*					
Amotivation	−3.9 (5.8)	−3.8 (5.4)	−3.2 (6.2)	−1.7 (3.8)	0.500
External regulation	−5.3 (5.3)	−5.6 (5.9)	−3.5 (4.4)	−6.3 (6.0)	0.415
Introjection regulation	−5.1 (3.2)	−5.6 (3.0)	−5.5 (2.9)	−4.7 (3.3)	0.752
Identified regulation	18.4 (7.0)	18.5 (5.8)	18.1 (6.6)	16.0 (6.0)	0.537
Integrated regulation	25.4 (12.5)	29.9 (10.6)	29.6 (7.7)	25.7 (8.8)	0.289
RAI (total sum score)	29.4 (22.8)	33.2 (19.3)	35.5 (20.3)	28.0 (17.2)	0.604

*Self-perceived health (ranked from 1 to 5, where in first question 1 represents excellent and 5 represents poor and in second question 1 represents much better now than one year ago and 5 represents much worse now than one year ago):*					
In general, would you say your health is:	3.2 (1.1)	2.9 (0.8)	2.9 (1.1)	3.2 (1.0)	0.619
Compared to one year ago, how would you rate your health in general now?	3.0 (0.8)	3.2 (1.0)	3.3 (0.8)	3.0 (0.9)	0.630

*Quality of life (ranked from 1 to 7, where 1 represents strongly disagree and 7 represents strongly agree):*					
In most ways my life is close to my ideal	5.2 (0.8)	4.3 (1.4)	4.7 (1.1)	4.0 (1.6)	0.017^*∗*^
The conditions of my life are excellent	5.3 (1.0)	4.3 (1.4)	4.9 (1.3)	4.4 (1.5)	0.153
I am satisfied with life	5.6 (1.3)	5.2 (1.3)	5.4 (0.9)	4.6 (1.7)	0.107
So far I have gotten the important things I want in life	5.5 (1.3)	4.8 (1.6)	5.3 (1.1)	4.7 (1.6)	0.198
If I could live my life over, I would change almost nothing	4.8 (1.7)	4.4 (1.6)	4.4 (1.5)	4.0 (1.6)	0.462

Total quality of life score	26.3 (5.2)	23.3 (6.7)	24.3 (4.9)	21.7 (7.0)	0.102

^*∗*^Statistically significant differences between groups at baseline.

**Table 3:** Primary and secondary outcome measures for completers in the different training groups A–C and control group D (*n* = 90) analyzed by one-way ANOVA at posttest. The results are shown as mean and standard deviation (SD).

Outcome measures	Group A	Group B	Group C	Group D	*p* value
	BodyPump (*n* = 23)	Personal trainer (*n* = 27)	Nonsupervised exercise (*n* = 19)	Control (*n* = 21)	
*Exercise motivation:*					
Amotivation	−2.7 (5.2)	−2.7 (5.7)	−0.3 (1.4)	−3.9 (6.8)	0.203
External regulation	−5.8 (6.6)	−3.7 (4.1)	−4.4 (4.9)	−7.1 (6.3)	0.182
Introjection regulation	−6.3 (3.1)	−4.7 (2.7)	−5.2 (3.0)	−4.1 (2.9)	0.065
Identified regulation	19.9 (4.7)	22.5 (5.1)	22.5 (5.7)	17.0 (5.9)	0.002^*∗*^
Integrated regulation	31.0 (8.3)	36.1 (7.7)	35.4 (10.1)	24.6 (8.9)	<0.001^*∗*^
RAI (total sum score)	43.9 (19.8)	47.6 (15.4)	48.4 (17.8)	26.5 (18.2)	<0.001^*∗*^

*Self-perceived health:*					
In general, would you say your health is:	2.7 (1.1)	2.7 (0.9)	2.5 (0.7)	2.9 (0.9)	0.690
Compared to one year ago, how would you rate your health in general now?	2.4 (0.8)	1.9 (0.8)	2.3 (0.8)	3.0 (0.6)	<0.001^*∗*^

*Quality of life:*					
In most ways my life is close to my ideal	4.7 (1.6)	4.6 (1.3)	5.0 (1.3)	4.3 (1.5)	0.513
The conditions of my life are excellent	5.0 (1.2)	5.0 (1.1)	5.1 (1.3)	4.7 (1.2)	0.656
I am satisfied with life	5.4 (1.4)	5.4 (1.0)	5.7 (1.0)	5.1 (1.3)	0.331
So far I have gotten the important things I want in life	5.0 (1.5)	5.4 (1.1)	5.5 (1.3)	5.1 (1.2)	0.439
If I could live my life over, I would change almost nothing	4.4 (1.4)	4.7 (1.5)	4.8 (1.0)	4.3 (1.7)	0.544

Total quality of life score	24.5 (11.3)	25.2 (5.1)	26.0 (4.6)	23.5 (6.1)	0.502

^**∗**^Statistically significant differences between groups at posttest.
